# Segmentation of dance movement: effects of expertise, visual familiarity, motor experience and music

**DOI:** 10.3389/fpsyg.2014.01500

**Published:** 2015-01-07

**Authors:** Bettina E. Bläsing

**Affiliations:** ^1^Faculty of Psychology and Sport Science, Neurocognition and Action Research Group, Bielefeld UniversityBielefeld, Germany; ^2^Center of Excellence - Cognitive Interaction Technology, Bielefeld UniversityBielefeld, Germany; ^3^Research Institute for Cognition and Robotics (CoR-Lab), Bielefeld UniversityBielefeld, Germany

**Keywords:** expertise, event segmentation, dance, movement learning, motor experience, music

## Abstract

According to event segmentation theory, action perception depends on sensory cues and prior knowledge, and the segmentation of observed actions is crucial for understanding and memorizing these actions. While most activities in everyday life are characterized by external goals and interaction with objects or persons, this does not necessarily apply to dance-like actions. We investigated to what extent visual familiarity of the observed movement and accompanying music influence the segmentation of a dance phrase in dancers of different skill level and non-dancers. In Experiment 1, dancers and non-dancers repeatedly watched a video clip showing a dancer performing a choreographed dance phrase and indicated segment boundaries by key press. Dancers generally defined less segment boundaries than non-dancers, specifically in the first trials in which visual familiarity with the phrase was low. Music increased the number of segment boundaries in the non-dancers and decreased it in the dancers. The results suggest that dance expertise reduces the number of perceived segment boundaries in an observed dance phrase, and that the ways visual familiarity and music affect movement segmentation are modulated by dance expertise. In a second experiment, motor experience was added as factor, based on empirical evidence suggesting that action perception is modified by visual and motor expertise in different ways. In Experiment 2, the same task as in Experiment 1 was performed by dance amateurs, and was repeated by the same participants after they had learned to dance the presented dance phrase. Less segment boundaries were defined in the middle trials after participants had learned to dance the phrase, and music reduced the number of segment boundaries before learning. The results suggest that specific motor experience of the observed movement influences its perception and anticipation and makes segmentation broader, but not to the same degree as dance expertise on a professional level.

## Introduction

Despite its continuous nature, human motor action is functionally based on task- and event related perception. Research suggests that ongoing processing resources are devoted to this perceptual process, and that the online perception of events determines how episodes are understood and encoded in memory (Zacks and Tversky, 2001; Kurby and Zacks, 2008). According to Event Segmentation Theory (Zacks et al., 2007), the perception of events depends on both sensory cues and knowledge structures that represent previously learned information about event parts and inferences about actors' goals and plans. Related studies have revealed that the segmentation of observed actions is crucial for the understanding and memorizing of these actions (e.g., Swallow et al., 2009; Zacks et al., 2009; Sargent et al., 2013). Furthermore, the theory states that any observed activity is spontaneously segmented into events during perceptual processing, which enables the system to anticipate upcoming information and react appropriately. As long as anticipation is successful, representations in working memory (named “event models” in this context, see Zacks et al., 2007; Kurby and Zacks, 2008) are maintained in a stable state, guiding further prediction and saving processing costs. When the frequency of anticipation errors increases as prediction becomes more difficult, event models are updated based on incoming information; these instances of increasing insecurity are subjectively experienced as boundaries between events. Perception of common goal-directed activities has been found to be hierarchical, with coarse-grained and fine-grained segmentation layers, corresponding to the hierarchical structure of action organization with goals and sub-goals. Furthermore, perception has been described as cyclical, with ongoing comparison of predictions to the perceived feeding back into processing. Event segmentation thereby results from the ongoing anticipation of what will happen next, which serves action understanding, prediction and learning.

Studies using event segmentation paradigms have shown that segmentation characteristics can be related to the understanding and memory of the observed actions. In these studies, actions from everyday life, such as assembling objects, setting a table or folding laundry, were presented to participants with no specified expertise (e.g., Zacks et al., 2009; Sargent et al., 2013). The presented actions typically involve the manipulation of objects and/or interactions between people, and are defined by clear action goals and a clear semantic context. In the context of dance or sports, the same characteristics do not necessarily apply. Even though many skilled actions in a sports context are object- and person-related and have clearly defined goals (e.g., passing the ball to a team member), there are also many other examples of movements that do not share these features. As such motor actions occur particularly in dance, the term “dance-like actions” has been used to describe motor actions that lack common features ascribed to actions from everyday contexts, such as interactions with objects and persons and obvious external action goals.

It has been stated that the goal of such dance-like actions is “the movement itself,” which certainly is often the case in a dance context. Schachner and Carey (2013) showed that observers tended to interpret actions as being intentionally movement-related if they were not able to infer external goals from observing the action, or if the action seemed to be inefficient or inappropriate with regards to any recognizable external goal. The authors state that a dance-like action is also (in the eye of the observer) primarily characterized by its goal, which is movement-based, whereas other “rational” actions have external goals. This is particularly true for dance movements, the goal of which is commonly not only movement-based but also related to communicating to partners or an audience via the body. It can therefore be assumed that segmentation of dance-like actions or dance movements follows different “rules” and cognitive strategies compared to segmentation of typical everyday activities with external goals.

Specifically in modern and contemporary dance, movement performance often requires a fluent quality that does not afford obvious partitioning or segmenting. The ability to perform long movement phrases with this obvious fluency is an important skill in these dance disciplines. This fluent quality of the movement can be supported and enhanced by the accompanying music or sound. Choreographers might choose music that does not have a clear beat or rhythm but that rather provides an associated sound layer, allowing the dancer and the spectator to integrate more freedom in integrating sound and movement. This means that the dance movement, when accompanied by music at all, does not necessarily follow a musical beat or rhythm, and might even contravene the music in order to create a more exciting impression for the audience. The interrelation between the dance movement and the accompanying music deliberately influences the spectator's perception and should therefore be taken into account when investigating the segmentation of dance movement; in this respect, dance differs from dance-like actions that are not commonly associated with music.

Numerous studies have provided evidence that the perception of skilled actions is modulated by expertise (see Cheung and Bar, 2012) and is specifically facilitated by motor experience of the observed action type (e.g., Abernethy and Zawi, 2007; Aglioti et al., 2008; Güldenpenning et al., 2011; Steggemann et al., 2011). Even though empirical approaches to expertise often differentiate between perception, cognition (e.g., decision making) and action (motor control), this distinction can hardly be maintained in the context of athletes' practice-dependent task-specific skills (see Yarrow et al., 2009). Evidence for the interdependency of perception, action and cognition in movement expertise has been found in many studies with athletes and other movement experts (e.g., Aglioti et al., 2008 see also Yarrow et al., 2009 for review). Dance expertise in particular has been shown to comprise a multitude of perceptual-motor and cognitive skills, including motor control, timing, learning, memorizing, imagery, entrainment, as well as multimodal communication and artistic expression (see Sevdalis and Keller, 2011; Bläsing et al., 2012; Waterhouse et al., 2014). Studies with expert dancers have shown that movement-related memory is more functionally structured in dancers than in non-dancers (Bläsing et al., 2009; Bläsing and Schack, 2012), and that dancers show shorter fixation times while watching dance movements than non-dancers, which points toward perception facilitation (Stevens et al., 2010). Furthermore, dance provides a highly adequate framework for studying expertise effects related to action-perception coupling, because dance, more than most types of sports, is performed with the primary goal of being observed by an audience. In dancers, increased activity has been found in specific brain areas commonly referred to as action observation network (AON) while watching familiar dance movements (Calvo-Merino et al., 2005). This network of brain regions (comprising the ventral and dorsal premotor cortices and parts of the parietal cortex, including the inferior parietal lobe, the superior parietal lobe, and the superior parietal sulcus, as well as the superior temporal sulcus) is typically involved in the execution, observation and imagery of actions. Studies showed that the activation of these regions is modulated differently by visual and motor expertise (Calvo-Merino et al., 2006). Dancers showed increased activity in areas belonging to the AON while watching movements from their own dance discipline compared to similar movements from other dance disciplines (Calvo-Merino et al., 2005; Cross et al., 2006). Activation of AON regions was further increased when dancers watched movements they had previously performed themselves, compared to movements they had frequently watched but not physically performed (Calvo-Merino et al., 2006). Learning to dance a specific movement phrase affects AON activation while watching the same phrase already early during the learning process (Cross et al., 2006). Different types of learning have been found to activate the AON in specific ways, with the right ventral premotor cortex responding specifically to the experience of having performed an observed movement, and the bilateral superior temporal cortex responding to the presence of a human model (Cross et al., 2009). These findings reflect that dance expertise affects both the production and the perception of dance-like movements. Dance expertise should therefore not only enable dancers to perform movement phrases fluently, but should also influence their perception of observed movement material in favor of fluency and greater over-all connectedness. Only few studies have investigated the segmentation of dance-like actions (e.g., Pollick et al., 2012; Noble et al., 2014), and so far none has focused on effects of dance expertise on segmentation. Evidence from preliminary studies suggests that observers' dance expertise affects the segmentation of dance-like actions, but not of other actions that have an obvious external goal (Bläsing et al., 2010).

The aim of the present study was to investigate how different factors, namely dance expertise, visual familiarity (via repeated presentations), motor experience (via learning to dance the presented phrase) and music would influence the segmentation of observed dance movement. Specifically, we presented a choreographed contemporary dance phrase of fluent character that did not contain interactions with objects or persons, communicative signals or semantic content. The dance phrase was choreographed on the basis of modern/contemporary dance technique, and was initially novel to all participants. This means that participants who regularly trained modern contemporary dance were likely to be familiar with the type of movement in general, but not with the presented movement material as such (note that modern/contemporary dance choreography commonly involves the exploration and creation of new movement material rather than re-combination and variation of defined partial movements, as this is often the case in classical dance). During the experimental procedure, the participants watched the sequence repeatedly and became thereby increasingly familiar with it. Their visual familiarity, in terms of knowing the exact dance phrase (rather than similar movement material from the same disciplinary background) was addressed here as a factor potentially affecting segmentation. It was expected that segmentation would become less variable with increasing visual familiarity over consecutive trials.

The issue of dance expertise was addressed in the current study by comparing groups of participants differing in their specific skill level in dance. In Experiment 1, professional dancers who had undergone professional dance training for many years and were currently all members of a professional dance theater company or free-lancing professional dancers performing with different companies as well as teaching dance on different levels (these participants are in the following referred to as “dancers”) were compared to sports students (in the following referred to as “non-dancers”) who had no particular experience in dance training apart from few very basic mandatory courses in their study program. It has to be pointed out that the non-dancer participants were “novices” only with respect to dance, but not to movement skills in general; most of them performed their preferred sports on an advanced to high level. For the purpose of the study, this group was preferred to a group of participants without any movement expertise (i.e., persons who did not perform sports or physical exercise on a regular basis) because of the specific segmentation task. Expertise has been shown to be task-specific and does not generalize well over domains (e.g., Ericsson and Charness, 1994). It was assumed that athletes without dance expertise would show similar responses to observed human body movement in general compared to dancers, including corresponding levels of motor activation and simulation, and that any differences in the results could be related to expertise in dance rather than a high level of physical training and motor skill in general. It was expected that dancers' segmentation behavior would differ from that of the non-dancers, with dancers defining less segment boundaries based on their training-based ability to anticipate dance movement more successfully despite its novelty, and their preference for viewing the observed dance movement as more connected.

As a third group, dance amateurs who trained modern/contemporary dance on intermediate level participated in Experiment 2 of the presented study. These participants (referred to as “amateurs” in the following) were chosen for two reasons. First, they represented a viable intermediate step between the non-dancers and the dancers, offering the opportunity to monitor expertise effects on different levels. Second, the amateur participants all belonged to the same dance class that was trained by the choreographer of the stimulus dance phrase. Crucially, this class was taught the dance phrase as part of their training schedule, which provided the opportunity to add the aspect of learning to that of expertise and relate the two aspects to each other. In Experiment 2, the participants thereby gained specific motor experience of the presented movement material (referred to as “motor experience” in the following, applied as factor in Experiment 2). The term “motor experience” is in this case related to the experience of having danced the exact phrase presented as stimulus, not more generally to experience with similar movement material from the same disciplinary background. It was expected that specific motor experience would increase the participants' expertise for the dance phrase and thereby make their segmentation behavior more “expert-like,” potentially even more than the dancers' in Experiment 1 who had greater dance expertise in general but no motor experience of the presented dance phrase.

As a fourth factor, the presence of music was added. The music chosen by the choreographer to accompany the dance phrase did not have a clear metric rhythm but rather consisted of an underlying sound layer of chords with slowly increasing and decreasing pitch and volume. It was hypothesized that the added music, because of its specific character, would influence the segmentation of the movement by reducing the number of segment boundaries, thereby binding movements together and reducing the over-all number of segment boundaries.

## Experiment 1: segmentation of a dance phrase by dancers and non-dancers: effects of visual familiarity and music

In Experiment 1, professional dancers and sport students without dance expertise repeatedly watched a video clip showing a dancer performing a phrase from a contemporary dance choreography. Each participant watched the sequence 20 times on a computer screen, 15 times without music followed by 5 times with music, and indicated segment boundaries by key press. This experiment was conducted in order to gain information about the effects of dance expertise, visual familiarity and music on the segmentation of dance movement.

### Method

#### Participants

Twenty-two participants voluntarily took part in Experiment 1 without any exchange for course credit or money. Twelve students of sport science (six females, one left-handed; age 25.91 ± 3.29 years, range 22–30 years) without any particular dance training experience (except for basic courses as part of their study program) were assigned to the non-dancers' group. All non-dancers were physically active; their most regularly performed sports included soccer, handball, rugby, and fitness training.

Ten professional dancers (six females, two left-handed; age 30.1 ± 6.59, range 23–40 years) participated as experts; all were trained in classical, modern and contemporary dance on professional level and were currently active as company dancers. Six of the dancers were current members of Tanztheater Bielefeld; four of the dancers were freelancing dancers and dance teachers.

All participants reported having normal or corrected-to-normal vision, and were naive with regard to the purpose of the experiment. All participants provided written informed consent before testing started. The experiment was performed in accordance with the ethical standards of the sixth revision (WMA, 2008) of the 1964 Declaration of Helsinki.

#### Apparatus and stimuli

The stimulus material consisted of a video clip (92 s, 2.290 frames, 25 Hz, recorded with a Sony camcorder) showing a dance phrase created and performed by dancer and choreographer Ilona Pászthy. The dance phrase was choreographed on the basis of modern/ contemporary dance technique, and was novel to all participants. For stimulus presentation and data collection, Interact® (Mangold) software running on a Notebook (Acer) with a 15 inch VGA-Display (vertical retraces 60 Hz) was used. The software recorded key presses during the presentation of the video clip, linked them to the adequate runtime and frame number and provided a protocol of these data.

#### Design and procedure

The data collection took place in a quiet lab or office room or in a free rehearsal space at the theater. Each participant was tested individually. During the experiment, the participant sat in front of the notebook computer and watched the presented video clip. The following instructions were given verbally by the experimenter: “You will now see a video clip of a dancer dancing a part of a dance piece. The clip will be repeated 20 times. While watching, please keep your finger on the space bar and press the space bar each time a part of the dance phrase ends and a new one begins. Apply your own criteria; you do not need to mark the same moments in each repetition.” This instruction was phrased in a similar way as instructions in previous segmentation studies (e.g., “to press a button… whenever… one natural and meaningful unit of activity ended and another began,” Zacks et al., 2009). No instruction was given regarding the resolution of segmenting (fine or coarse), as had been done in other segmentation studies (e.g., Swallow et al., 2009; Zacks et al., 2009). The sequence was presented 20 times, the first 15 trials without sound, followed by five trials accompanied by the music that had been chosen by the choreographer.

After completing all 20 trials, the participant was verbally asked two questions by the experimenter:

1. Which criteria or strategies did you use for segmenting the dance phrase?
2. Did the music in the last five trials affect your decisions?

The answers were written down by the experimenter in the form of key notes. This explorative interview was not carried out according to any established qualitative method, but was added to the data collection only to gain an impression of the participants' use of criteria and strategies. It was not expected that participants would be able to give a complete and objective account of their segmenting behavior, but the experimenter was rather interested in the criteria and strategies the participants applied explicitly or even deliberately. The complete experimental session for each participant lasted 60–90 min.

#### Data analyses

For every participant, the number of segment boundaries was recorded for each of the 20 trials. Mean group results of dancers and non-dancers were calculated for each trial number (1–20) separately, for all trials together, and for four groups of trials (trials 1–5: early trials; these trials were regarded as familiarization phase during which visual familiarity with the dance phrase was still low; trials 6–10: middle trials, with increasing visual familiarity; trials 11–15: late trials; for these trials, visual familiarity with the dance phrase was regarded as high; and trials 16–20: music trials, presented with sound). Non-parametric tests were applied to compare dancers and non-dancers regarding their defined numbers of segment boundaries for each trial separately, for all trials, and for each group of five trials (early trials, middle trials, late trials and music trials). Within each group of participants, mean numbers of segment boundaries of the four trial groups (early, middle, late, and music) were compared to each other using non-parametric tests (Mann Whitney *U*-test, Wilcoxon signed-rank test).

### Results

#### Segment boundaries

**Figure 1:**
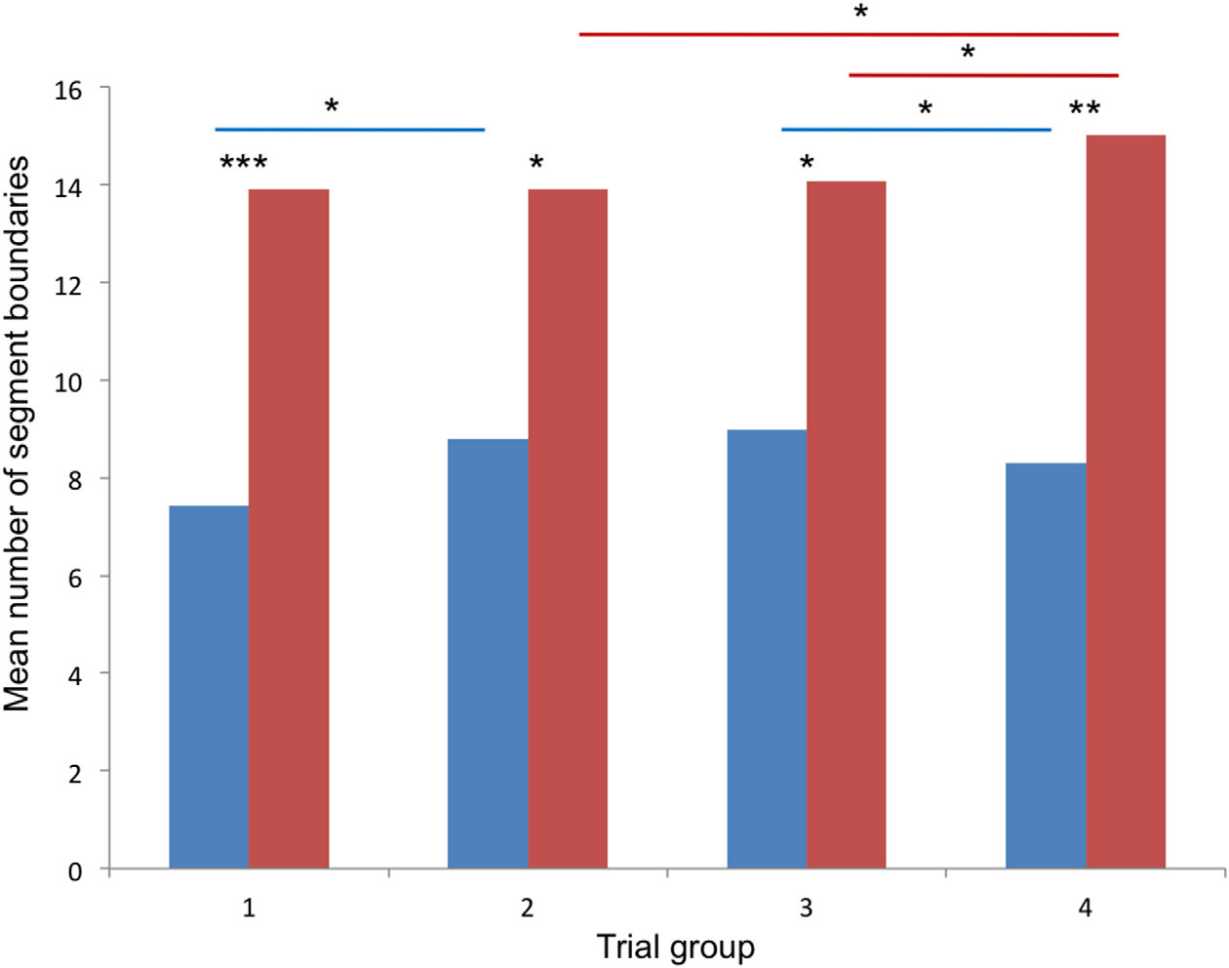
**Results of Experiment 1**. Mean numbers of segment boundaries defined for the four groups of trials [1: trials 1–5 (early); 2: trials 6–10 (middle); 3: trials 10–15 (late); 4: trials 16–20 (music)]; blue columns: dancers, red columns: non-dancers; asterisks mark significant differences: ^*^p < 0.05, ^**^p < 0.01, ^***^p < 0.001.

Comparisons between dancers and non-dancers (Mann-Whitney *U*-test) revealed that dancers generally defined less segment boundaries than non-dancers for all trials together (*z* = −2.853, *p* = 0.005), for each individual trial (trials 1–5: *p* < 0.01; trials 6–13: *p* < 0.05; trials 14–20: *p* < 0.01), and for all groups of trials (early trials: *z* = −3.269, *p* = 0.001; middle trials: *z* = −2.474, *p* = 0.013; late trials: *z* = −2.440, *p* = 0.015; music trials: *z* = −2.969, *p* < 0.003). Comparisons between groups of trials (Wilcoxon signed-rank test) in the non-dancers revealed differences between middle trials and music trials (*z* = −2.296, *p* = 0.022) and between late trials and music trials (*z* = −2.173, *p* = 0.030), with more segment boundaries occurring in the music trials than in the other groups. In the dancers, less segment boundaries were defined in the early trials than in the middle trials (*z* = −2.018, *p* = 0.044). In contrast to the non-dancers' results, less segment boundaries were defined in the music trials than in the late trials (*z* = −2.092, *p* = 0.036). Results for the four groups of trials are displayed in Figure 1, results for all individual trials are shown in **Figure 3**. The distribution of segment boundaries (calculated as average over all trials for 92 bins of 1 s) as defined by the experimental groups is illustrated in **Figure 4**.

#### Post-hoc interviews

After finishing the experimental procedure, each participant was asked two questions:

1. Which criteria or strategies did you use for segmenting the dance phrase?
2. Did the music in the last five trials affect your decisions?

The experimenter asked the participant verbally and wrote down the answers in key points (note that this informal procedure did not follow any established qualitative approach but only aimed at gaining additional information in an explorative way).

From the informal answers to Question 1, the most common criteria were extracted, and naming frequencies of these criteria were counted. The most common criteria and their frequencies of naming are displayed in Table 1. Remarks made by individual participants in response to Questions 1 and 2 are listed in Supplementary Table 1.

**Table 1:** **Segmentation criteria named by the three groups of participants in the *post-hoc* interviews (numbers indicate absolute frequencies of naming in both experiments)**.

**Segmentation criteria**	**Non-dancers**	**Amateurs**	**Dancers**
Change of movement type	7	8	4
Change of height level	3	3	6
Stops, pauses	5	2	3
Change of direction in space	4	2	3
Change of main active body part	4	1	3
Change of tempo, dynamics	–	8	5
Feeling, imagery	–	5	4
Movement impulse, accents	–	3	2
Cues for learning or teaching	–	5	–
Change of energy or force	–	–	4

### Discussion

In Experiment 1, dancers and non-dancers segmented a dance phrase repeatedly presented in a video clip by key press. Segmentation grain (i.e., numbers of segment boundaries) was expected to be influenced by expertise (comparison between the two groups), by visual familiarity of the movement phrase (comparison between early, middle and late trials), and by music (comparing the last group of trials presented with music to the previous groups of trials).

The results showed that in all trials, in each individual trial and in the four groups of trials, dancers generally defined less segment boundaries than non-dancers. The effect of expertise on movement segmentation was thereby very clearly reflected by the results, with dancers defining less segment boundaries and thereby segmenting the whole movement phrase into fewer and longer sections than non-dancers. This finding is supported by the comment of one dancer, who reported perceiving the entire phrase as a whole, “in a flow,” therefore segmenting did not feel natural. Perceiving a longer dance phrase as a whole despite the occurrence of various movement characteristics that could be used (and were typically named) as segmentation criteria is also in accordance with the claim often made in modern and contemporary dance to dance longer phrases fluently without obvious breaks or partitions, without “losing the energy.” The finding that this principle commonly applied to the dancers' action performance is transferred to perception when observing a dance phrase accords with the principle of perceptual resonance (Schütz-Bosbach and Prinz, 2007) described in various areas of expertise (e.g., Kiesel et al., 2009; Güldenpenning et al., 2011; Steggemann et al., 2011). Dancers defined less segment boundaries in early trials than in the middle and late trials, whereas no difference between early, middle and late trials was found in the non-dancers. An effect of visual familiarity was thereby only found in the dancers, but not in the non-dancers.

Interestingly, music affected segmentation differently in dancers and non-dancers: In the music trials, dancers defined less segment boundaries than in late trials, whereas non-dancers defined more segment boundaries in music trials than in middle and late trials. Apparently, music had a binding effect on the perceived movement in the dancers' group. (Comments given by individual dancers in response to Question 2 supported this interpretation: music was experienced as binding the movement together, slowing down the movement, adding a harmonic feeling). In the non-dancers, in contrast, music seemed to confuse and thereby cause more segment boundaries to occur, possibly based on the perceived lack of segmentation cues in the music that might have interfered with previously defined movement cues.

Expertise in sport or dance typically involves visual as well as motor experience of specific actions, and differences found between experts and novices can be based on any of the two, or both. To gain further understanding of expertise effects in action perception, it is necessary to differentiate visual and motor expertise either by studying observation experts (e.g., Calvo-Merino et al., 2006; Aglioti et al., 2008) or by applying a learning intervention (e.g., Cross et al., 2006, 2009). In order to gain information about potential effects of motor experience on segmentation, a second experiment was conducted with dance amateurs who solved the same experimental task as applied in Experiment 1 before and after learning the presented dance phrase.

## Experiment 2: segmentation of a dance phrase before and after learning: effects of motor experience, visual familiarity and music

In Experiment 2, the same segmentation task as in Experiment 1 was applied to dance amateurs who regularly trained modern dance in the same group. After learning the phrase in their training as part of a performance program, all participants repeated the experimental task. The main goal of the experiment, apart from adding a third (intermediate) group of participants, was to gain information regarding the effect of specific motor expertise on segmenting a dance phrase.

### Method

#### Participants

Eight participants (all female, one left-handed; age 18.5 ± 6.55 years, range 14–30 years) voluntarily took part in Experiment 2 without any exchange for course credit or money. All participants trained regularly in classical and contemporary dance on average to advanced amateur level (years of training in classical dance: 9.13 ± 1.45 years, range: 8–12 years; years of training in modern dance: 3.38 ± 1.51 years, range: 1–6 years; dance training: 3.38 ± 2.20 h per week, range: 2–6 h) and were currently members of the same modern dance class (Theater Bielefeld ballet school). All eight participants named classical and modern dance as their primary types of training, single participants also trained in one of the following disciplines: capoeira, hip-hop, karate and acrobatics. All participants reported having normal or corrected-to-normal vision, and were naive with regard to the purpose of the experiment. All participants provided written informed consent before testing started. The experiment was performed in accordance with the ethical standards of the sixth revision (WMA, 2008) of the 1964 Declaration of Helsinki.

#### Apparatus and stimuli

The same stimulus material and experimental set-up was used as in Experiment 1.

#### Design and procedure

Two data collections were applied, one before and one after the participants learned the dance phrase in their training. Data collections took place in a quiet lab or office room or in a free dress room at the ballet school. Each participant was tested individually, the experimental procedure was exactly the same as in Experiment 1. The single experimental session lasted 60–90 min.

After all participants had completed the experiment once (data collection 1, pre-learning), they learned the presented dance phrase as part of their regular training. After approximately 6 weeks in which the dance phrase had been trained regularly, the experiment was repeated in exactly the same way as before (data collection 2, post-learning). Crucially, at the time of data collection 1, participants were neither informed that they would learn the dance phrase nor that they would be asked to participate in a second data collection, and participants were not informed about data collection 2 when learning the dance phrase. The data collections were separated by a time interval of approximately 6 weeks during which the dance phrase was learned and trained as part of a choreography for later stage performance.

#### Data analyses

Mean numbers of segment boundaries were analyzed in the same way as for Experiment 1. Non-parametric tests were applied to compare the two experimental conditions, pre- and post-learning (i.e., without and with motor experience of dancing the phrase, respectively), regarding the defined numbers of segment boundaries for each trial separately, for all trials, and for each group of five trials (early trials, middle trials, late trials, and music trials). For each data collection (pre- and post-learning), mean numbers of segment boundaries of the four trial groups (early, middle, late, and music) were compared to each other using non-parametric tests (Wilcoxon signed-rank test).

### Results

#### Segment boundaries

**Figure 2:**
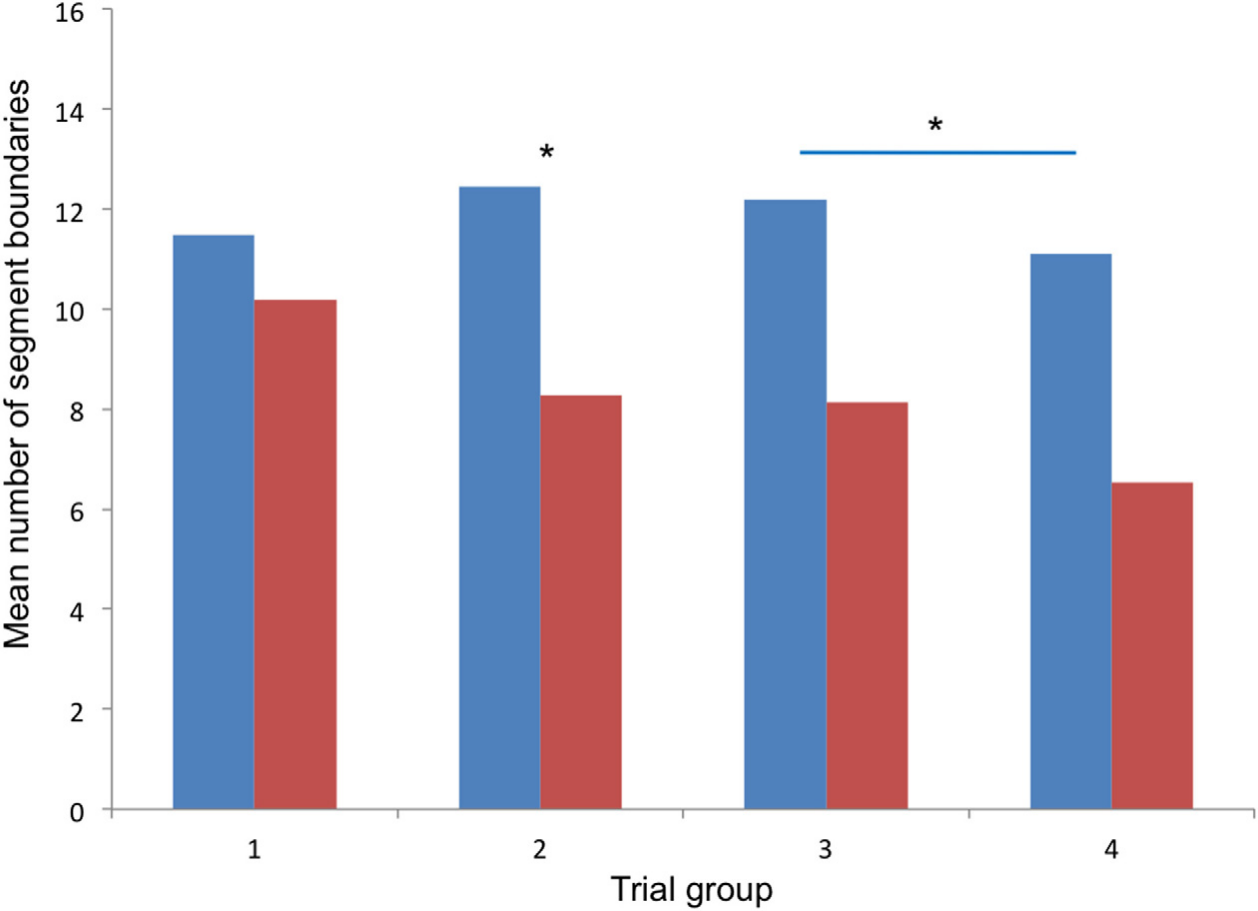
**Results of Experiment 2**. Mean numbers of segment boundaries defined for the four groups of trials (1, early trials; 2, middle trials; 3, late trials; 4, music trials); blue columns: before learning, red columns: after learning; asterisks mark significant differences: ^*^p < 0.05.

**Figure 3:**
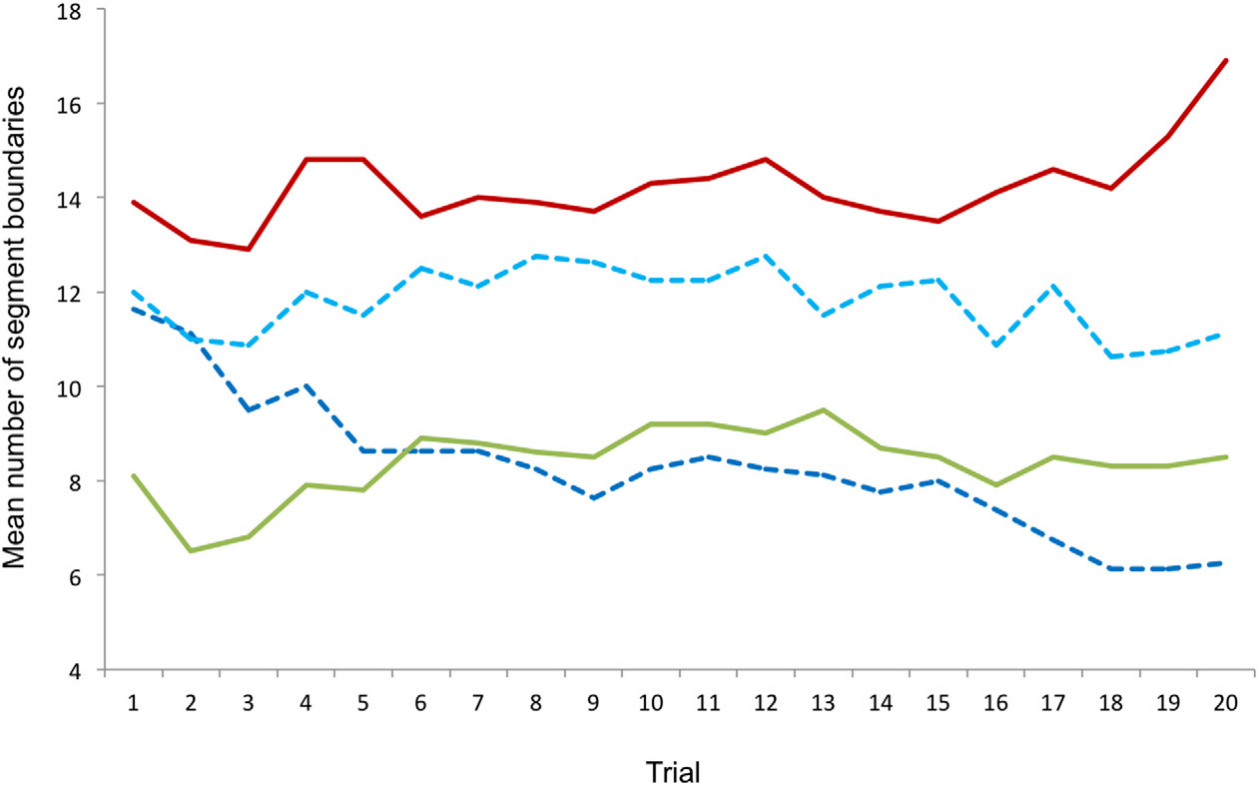
**Results of Experiment 1 (full lines) and Experiment 2 (dashed lines) combined, mean numbers of segment boundaries defined for individual trials**. Red, non-dancers; green, dancers; light blue, amateurs before learning; dark blue, amateurs after learning.

**Figure 4:**
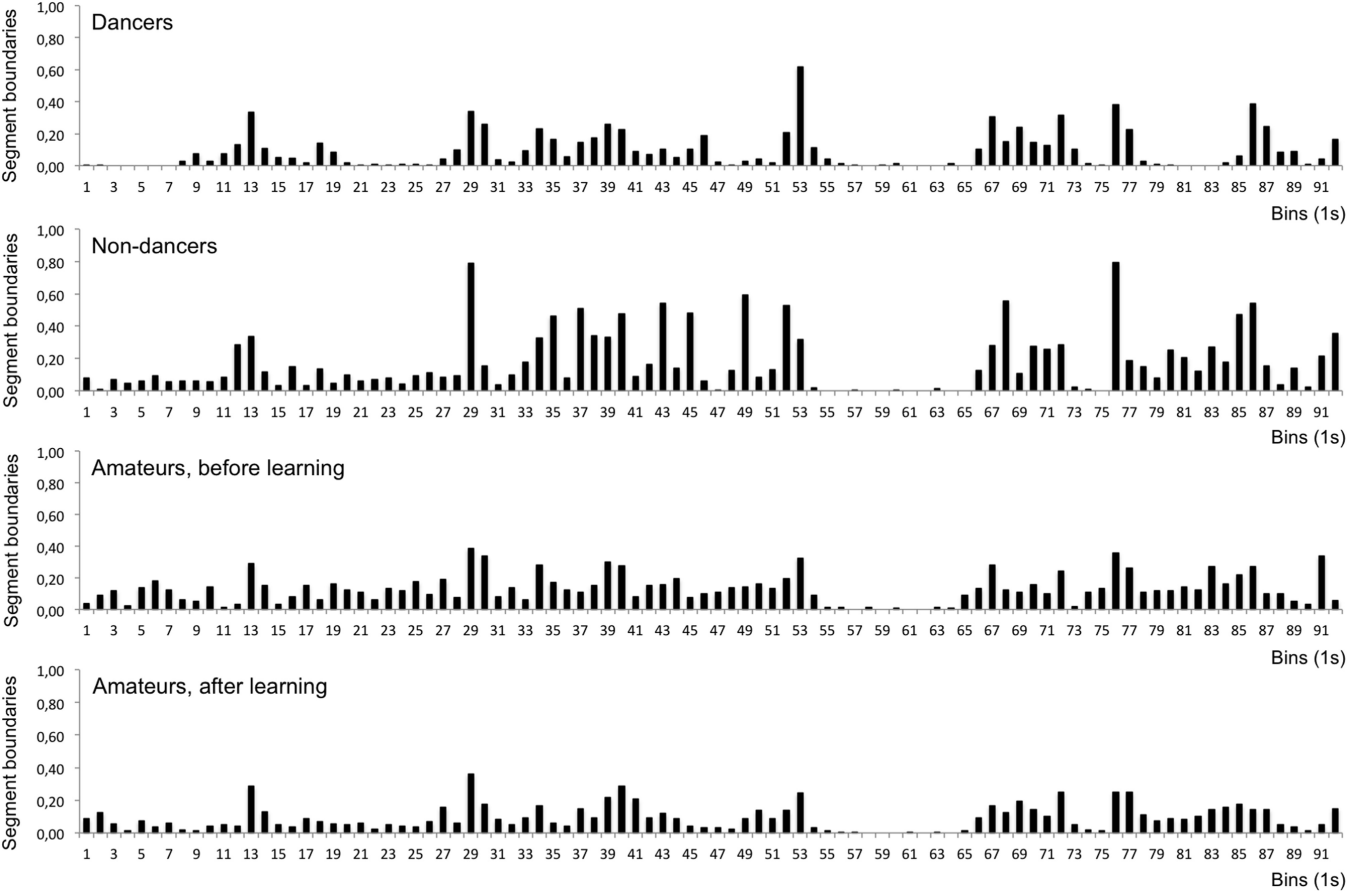
**Results of Experiments 1 and 2**. Distribution of segment boundaries over the phrase: average number of segment boundaries for each bin (1 s), calculated over all trials; panels from top to bottom: dancers, non-dancers, amateurs before learning the phrase, amateurs after learning the phrase. The most frequently set segmentation boundaries marked the following instances in the dance phrase: 13 change of speed and direction during a floor sequence; 29 slowing down and raising to the knees from floor sequence; 53 slowing down floor movement, raising into shoulder stand; 76 transition from walking to dynamic swinging movement initiated by an impulse of the right shoulder; 86 going down to the floor from standing.

Comparisons of trial groups between the pre- and post-learning conditions (Wilcoxon signed-rank test) revealed a difference in the middle trials (*z* = −2.240, *p* = 0.025), in which less segment boundaries were defined in the post-learning condition than in the pre-learning condition. Comparisons of individual trials revealed differences in trials 8, 9, 10, 12, and 15 (all *p* < 0.05). No difference between pre- and post-learning was found, however, when comparing segment boundaries over all trials. Comparisons between groups of trials within each condition revealed that only in the pre-learning condition, less segment boundaries were defined in the music trials than in the late trials (*z* = −2.371, *p* = 0.018), whereas trial groups did not differ in the post-learning condition. Results for the four groups of trials are displayed in Figure 2, results for all individual trials (Experiments 1 and 2) are shown in Figure 3. The distribution of segment boundaries (calculated as average over all trials for 92 bins of 1 s) as defined by the amateurs before and after learning the dance phrase is illustrated in Figure 4.

Comparing the group of amateurs to the groups of dancers and non-dancers from Experiment 1 showed differences between the non-dancers and the amateurs in the pre-learning condition for the early trials (*z* = −2.013, *p* = 0.044) and the music trials (*z* = −2.394, *p* = 0.017), and differences between the non-dancers and the amateurs in the post-learning condition in all groups of trials (early: *z* = −2.355, *p* = 0.019; middle: *z* = −2.431, *p* = 0.015, late: *z* = −2.546, *p* = 0.011, music: *z* = −3.009, *p* = 0.003), as well as over all trails (*z* = −2.508, *p* = 0.012). No differences were found between the dancers' and the amateurs' results. Results for all individual trials are displayed in Figure 3.

#### Post-hoc interviews

As in Experiment 1, each participant was verbally asked two explorative questions after each data collection (again, no established qualitative approach was applied but the two questions were asked informally and key points of the answers were written down by the experimenter). The most common criteria and their frequencies of naming (in response to Question 1: “Which criteria or strategies did you use for segmenting the dance phrase?”) are displayed in Table 1. Remarks made by individual participants in response to Questions 1 and 2 (“Did the music in the last five trials affect your decisions?”) are listed in Supplementary Table 1.

### Discussion

In Experiment 2, dance amateurs segmented a dance phrase repeatedly presented in a video clip by key press. The experiment was repeated after the participants had learned to dance the presented dance phrase. Segmentation grain (i.e., numbers of segment boundaries) was expected to be influenced by motor experience (comparison between pre- and post-learning), by visual familiarity of the movement phrase (comparison between early, middle and late trials), and by music (comparing the last group of trials presented with music to the previous groups of trials).

Results showed that less segment boundaries were defined in the post-learning condition compared to the pre-learning condition in the middle and late trials. No difference between pre- and post-learning was found in the early trials and in trials with music, and when comparing mean numbers of segment boundaries over all trials. Consequently, the motor experience of dancing the presented movement phrase was found to affect segmentation grain slightly, with less segment boundaries being defined by the participants after they had learned to dance the phrase, however, this difference only reached significance in the middle and late trials. This finding was reflected by the participants' comments: segments were perceived as longer, “larger shapes” were recognized, longer segments were “more fun.” Participants' comments also reflected that watching the dance phrase was experienced as more embodied and more competent after learning.

Music was found to affect segmentation in the pre-learning condition, but not in the post-learning condition. Before learning the dance phrase, less segment boundaries were defined in the music trials than in the late trials, showing an effect of music on segmentation comparable to the one observed in the dancers. It can be assumed that the effect was caused by a binding effect of music (as assumed in the dancers). The finding that the difference between late trials and music trials was not significant anymore after learning the movement phrase might be explained by the fact that the participants danced the phrase in their training in combination with the same music as used in the experiment, therefore music and movement might have been coupled during the learning and training process. When watching the movement without the music in the post-learning condition, participants did not really experience the movement “without the music,” as music and movement had become parts of the same integrated representation in their long-term memory (see Land et al., 2013), and segmentation (even when no music was played) related not only to the presented movement, but to the representation of “movement-with-music.”

In contrast to Experiment 1, no differences were found between early, middle and late trials within each condition, showing that no effect of visual familiarity was found in the amateurs (or that the potential effect of visual familiarity was too weak to produce significant results). The finding that visual familiarity did not significantly affect segmentation is contrasted by the impression of several participants that, with repeated observations of the dance phrase in consecutive trials, the dance phrase was more strongly perceived as a whole (the movement was “growing together”). Similar to the dancers, several amateurs had expressed before learning that they found it difficult to segment the phrase because of the perceived fluency and connectedness of the movement.

Comparing the results of the amateurs' group in Experiment 2 to the results of the two groups of participants from Experiment 1 revealed that the amateurs did not differ from the dancers, whereas differences found between the amateurs and the non-dancers were found and increased from the pre-learning to the post-learning condition. These findings indicate that the amateurs might have become more “expertly” in perception and segmentation by learning to dance the presented movement, which corroborates findings on effects of motor expertise on action perception (e.g., Aglioti et al., 2008; Güldenpenning et al., 2011).

### General discussion

Expertise in dance and various sports disciplines has been found to modulate the perception of actions on different levels, specifically of those actions belonging to the specific area of expertise (Calvo-Merino et al., 2005, 2006; Aglioti et al., 2008; Cheung and Bar, 2012). Evidence exists that these effects specifically relate to motor experience and learning of the observed actions (Cross et al., 2006, 2009). Furthermore, Event Segmentation Theory (Zacks et al., 2007) and related empirical studies (e.g., Zacks et al., 2009; Sargent et al., 2013) provide evidence for the assumption that the segmentation of observed actions is influenced by relevant visual and motor expertise. In the present study, this assumption was tested using segmentation of a dance phrase as experimental task performed by three groups of participants differing in dance expertise. Results of two consecutive experiments revealed broader segmentation applied by professional dancers, but also by dance amateurs, compared to non-dancers. The effect was increased in the amateurs after learning the presented dance phrase, pointing toward a specific effect of motor experience. It has to be emphasized that in this study participants were not instructed to segment with fine or coarse segmentation grain, whereas this was commonly done in studies on event segmentation. When participants were instructed to segment observed actions into coarse units, this typically resulted in segment lengths of 30–60 s, whereas the instruction to apply fine-grained segmentation resulted in units of 10–20 s. Zacks et al. (2009) showed that fine-grained segmentation generally correlated with movement parameters whereas coarse-grained segmentation rather correlated with external goals and context information. In the current study, average segment lengths defined by the participants ranged between 6 and 13 s. As the context of the presented movement in this study was dance and the dancer's intention was clearly movement-related, it can be assumed that segmentation was predominantly fine-grained, and the results support this assumption.

Visual familiarity was induced in the current study by repeated presentation of the same stimulus dance phrase. This approach clearly differs from the approaches taken in other segmentation studies in which observes typically watched the same action twice, in part with different instructions (e.g., coarse vs. fine segmentation, Zacks et al., 2009; Sargent et al., 2013; or just watching vs. segmenting, Noble et al., 2014). Under natural conditions, actions are not repeated in exactly the same way, and segmentation occurs spontaneously as part of perceptual processing. The same commonly applies to watching dance: most audience members watch the performance of a dance piece once, from a naive perspective. “Expert observers” like dancers, choreographers, teachers, and dance enthusiasts watch the same movement phrases repeatedly, however, performances naturally differ. Watching different versions of the same movement material performed with natural variation may increase the observers' sensitivity for subtle differences. Watching the identical performance 10 or 20 times (e.g., from a video clip) certainly increases the observer's sensitivity for details, but might also become boring. In the current study, the phrase was presented repeatedly to create high visual familiarity with the previously unknown phrase, thereby facilitating prediction and increasing anticipation success. Following Event Segmentation Theory (Zacks et al., 2007), this should result in a decrease of the number of segment boundaries. Results of this study confirm this assumption, and individual participants' comments reflect this (see Supplementary Table 1). Other participants' comments suggest that certain segment boundaries became fixed over trials, as participants felt more confident to be “right” in their decisions. This aspect of experiencing competence might play a crucial role for the esthetic evaluation of dance, which has been related to prediction success and moments of surprise (e.g., Hagendoorn, 2004). Studying segmentation in relation to the novelty and esthetic evaluation of dance movement would be highly relevant in this context, addressing for instance observers' subjective experiences of boredom and competence. In the present study, several participants reported that they deliberately varied their strategies, playfully trying out different ways of segmenting (see Supplementary Table 1). This creative attitude toward the experimental task might have been an attempt to counteract boredom, but might also have been elicited by the type of stimulus (dance) and the general appreciation of watching it.

Music added during the last five trials had contradictory effects in the different experimental groups. While music decreased the number of segment boundaries in the dancers and in the amateurs (before learning), it increased the number of segment boundaries in the non-dancers. This finding has been interpreted in terms of a binding effect in the dancers and amateurs and irritation in the non-dancers.

Empirical evidence exists that event segmentation not only occurs while observing actions, but also while listening to music (Sridharan et al., 2007). Based on the proposed multimodality of event models (see Zacks et al., 2007), it can be assumed that music modifies the perception (and performance) of the dance movement it accompanies, by potentially increasing the experience of uncertainty in movement prediction at musical transition points (Sridharan et al., 2007) and decreasing it within musical phrases. The effect of this interrelation between perceived movement and sound on segmentation is likely to depend on the specific characteristics of both and their temporal relation to each other. As listening to music alone has been shown to be sensitive to event segmentation (Sridharan et al., 2007), it can be assumed that music would affect the segmentation of movement in different ways, depending on its characteristics (i.e., its metrics, pitch, rhythm, pulse, etc.). In the current study, the music that accompanied the movement did not have any metric rhythm or pulse, but consisted of slowly rising and falling chords, and might therefore have had a binding rather than a dividing influence. It can furthermore be hypothesized that segmentation of dance movement accompanied by music would be sensitive to the way both are integrated, and to what extent the dancer entrains with the music, reacts to musical cues or deliberately counterpoints them. This aspect and the more general question to what extent music influences the segmentation of observed dance movement warrants further study. It would be particularly interesting to systematically combine movement and sound in different ways to shed light on the roles of visual and auditive information and their interrelation for the perception and segmentation of dance-like actions. Research responding to these questions would not only be of interest for scientists investigating action perception, but also for dancers and choreographers interested in audience reactions. Promising manipulations could include the presentation of a movement phrase combined with different types of music and sound, or with the same music or sound varying in temporal relation (i.e., music systematically shifted relative to the movement). In such studies, the different combinations of music and movement would have to be presented in a counterbalanced way to control for order effects. In the current study, this was not the case; music was added only to the last five trials to prevent interference with the factor visual familiarity. Confounding of the factor music with visual familiarity can therefore not be excluded, which represents a clear limitation of the current study with regards to the influence of music on movement segmentation. Furthermore, as previously mentioned, the typical situation of a dance spectator is to watch a dance performance once, without knowing it. Therefore, potential effects of music on the perception of novel, unfamiliar and potentially surprising movement material would be relevant to study in the context of dance.

The task of parsing movement phrases has also been applied as artistic tool in choreography, as it is assumed to have the potential to change the dancers' perception of the movement and thereby their artistic expression. In a “parsing and viewing” task performed by Wayne McGregor | Random Dance as part of a choreographic process, dancers segmented movement phrases and subsequently commented their decisions, which revealed different cognitive frameworks underlying dance parsing (deLahunta and Barnard, 2005). To attend to the latter aspect in our study, the participants of the current study were asked informally about their personal segmentation criteria and strategies. Participants of all groups named changes in movement characteristics (movement type, active body part, height level, direction, speed). Criteria related to learning the movement were only named by the amateurs (e.g., “how the teacher would teach it”), whereas only the dancers named dynamic features (e.g., “where force would be needed”). It can be assumed that the latter criterion requires efficient on-line motor simulation of the observed movement, which might be a skill that is specific to dancers on a high level of expertise (see Bläsing and Schack, 2012). To address this issue, further research would be needed relating experts' segmentation with movement analysis. Similar approaches have been used by Zacks et al. (2009) for actions from a non-dance context and by Noble et al. (2014) for Indian dance with a narrative character, but these studies did not include an expertise-related paradigm. It would be of particular interest to know to what extent dancers, compared to non-dancers, are able to specify and predict dynamical measures (i.e., forces) from motor simulation while watching dance movement, and how this influences the way they segment a dance phrase.

An aspect of this study that has been rarely addressed is the combination of comparing expertise with a learning task. Learning, however, was investigated only in the amateur group with intermediate skill level, but not on different levels of expertise, which could be a topic for further study. A related question of interest that could not be addressed here is to which extent the way movement material is learned or taught affects the perception and segmentation of the same material later on. In other words, would the way the teacher has structured the dance phrase be reflected by the segment boundaries defined by the students after learning the phrase? In dance training, teachers commonly break movement phrases down into sub-phrases and partial movements in order to facilitate learning, and students imitate and practice these parts and subsequently combine them again to a whole phrase. This procedure naturally affords breakpoints in the mental representation of the phrase in the dancer's memory that can become undesired breakpoints in the fluent quality of movement performance. To cure this problem, different measures are taken during further training of the phrase, such as varying the lengths of partial phrases and paying special attention to the transitions. Studies using segmentation paradigms could help to shed light on the effects and the efficiency of such practice.

Taken together, it can be concluded that segmentation of dance movement is clearly influenced by expertise, with broader segmentation grain being applied by professional and amateur dancers than non-dancers. Effects of visual familiarity and music on movement segmentation seem to be modulated by expertise, and motor experience had a slight effect in the amateur dancers. These findings contribute to the literature on dance expertise and segmentation of dance-like actions, and raise future research questions particularly addressing effects of the novelty or familiarity of the observed movement material, interrelations between movement and music or sound, learning in different ways and on different levels of expertise, and the esthetic evaluation of the observed dance movement.

### Conflict of interest statement

The author declares that the research was conducted in the absence of any commercial or financial relationships that could be construed as a potential conflict of interest.
