# Supplementary material for: Segmentation of dance movement: effects of expertise, visual familiarity, motor experience and music
Source: Front Psychol. 2015 Jan 7;5:1500. doi: 10.3389/fpsyg.2014.01500 (PMC4285866; doi:10.3389/fpsyg.2014.01500)
Supplement: Supplementary file 1 [file Table1.DOCX]

**Appendix**

Supplementary Table 1: Key notes taken by the interviewer during the explorative informal interviews

| Question 1: Which criteria or strategies did you use for segmenting the dance phrase? | | |
| --- | --- | --- |
| Dancers | | counting as a strategy for segmenting was tried, but did not work;  criteria changed during the experiment;  verbal phrasing was used to mark segments;  the entire phrase was "in a flow", perceived as a whole, therefore segmenting was difficult and did not feel natural;  beginnings of new segments were easier to define than end points;  segment boundaries became more fixed and constant after several trials;  segment boundaries were defined where force would be needed when mentally dancing the movement. |
| Non-dancers | | criteria changed during the experiment;  segment boundaries became more fixed and constant after several trials;  verbal phrasing was used to mark segments. |
| Amateurs,  before learning | | segmentation was based on the imagination of dancing the movement and on the perceived intensity of feeling the movement;  not all breaks marked relevant segment boundaries;  segmenting became more and more difficult, as the movement was somehow "growing together" over trials;  number of segment boundaries decreased over trials, it became harder to recognize segment boundaries at all;  how the teacher would teach the movement, or "how I would teach myself";  the movement was compared to the teacher's other movement material;  the entire movement was in one flow, there were no real segment boundaries;  different ways of segmenting were tried out: first according to impulse, later according to changes in movement type, direction or tempo (the latter produced shorter segments);  segmenting became easier over trials, and segments became longer;  segment boundaries were defined "when a new movement could start", "when I felt insecure about what movement came next"; "when I did not know how it continued". |
| Amateurs,  after learning | | segmenting was different from the first time, clearer and easier;  less segment boundaries were defined (i.e., segments were longer) than the first time;  segmenting was more difficult, even though the movement was familiar;  "I knew what to expect, knew the movement, knew where to make breaks or change the dynamics";  "I danced the movement in my head while watching, I even felt my body move while watching";  "The first time I segmented based on emotions. Now I segmented differently because I knew how it would continue";  "I had an internal feeling of pausing ("innehalten") that showed me where movements finished";  "I recognized larger shapes than the first time, for example now I realized that there was a circle, whereas the first time I saw only the steps";  "I tried different ways of segmenting and found that larger segments were more fun, smaller segments were rather boring". |
| Question 2: Did the music in the last five trials affect your decisions? | | |
| Dancers | music was irritating or distracting in the first music trial(s), but not later on;  music did not affect segmenting because there were no clear rhythmic features or accents in the music;  music might have influenced segmentation slightly, but it was unclear in which direction;  music confirmed or strengthened previously selected segment boundaries;  music bound movement together, therefore less segment boundaries were defined;  music was perceived as slowing down ("rallentando") the observed movement;  music added a harmonic feeling to the movement. | |
| Non-dancers | music was expected to make segmenting easier, but this was not the case;  music was irritating or distracting in the first music trial(s), but not later on;  music did not affect segmenting because there were no clear rhythmic features or accents;  music might have influenced segmentation slightly, but it was unclear in which direction. | |
| Amateurs,  before learning | music did not help, distract or irritate;  music did not change segmenting, as it fit well with the movement and did not affect movement expression;  music was very irritating and made segmenting more difficult, by raising questions about choreography (e.g., was the music supposed to cause movement impulse or not?);  music was irritating or confusing at first, but not later on;  segment boundaries were marked when the musical chord or key changed. | |
| Amateurs,  after learning | music confirmed previously selected segment boundaries;  music clearly defined movement phrases;  music made segments longer;  music marked climax points in the movement. | |
